# Regulation of neuritogenesis in hippocampal neurons using stiffness of extracellular microenvironment

**DOI:** 10.1371/journal.pone.0191928

**Published:** 2018-02-06

**Authors:** Aya Tanaka, Yuki Fujii, Nahoko Kasai, Takaharu Okajima, Hiroshi Nakashima

**Affiliations:** 1 NTT Basic Research Laboratories NTT Corporation, Atsugi, Kanagawa, Japan; 2 Graduate School of Information Science and Technology, Hokkaido University, Sapporo, Hokkaido, Japan; Emory University/Georgia Institute of Technology, UNITED STATES

## Abstract

The mechanosensitivity of neurons in the central nervous system (CNS) is an interesting issue as regards understanding neuronal development and designing compliant materials as neural interfaces between neurons and external devices for treating CNS injuries and disorders. Although neurite initiation from a cell body is known to be the first step towards forming a functional nervous network during development or regeneration, less is known about how the mechanical properties of the extracellular microenvironment affect neuritogenesis. Here, we investigated the filamentous actin (F-actin) cytoskeletal structures of neurons, which are a key factor in neuritogenesis, on gel substrates with a stiffness-controlled substrate, to reveal the relationship between substrate stiffness and neuritogenesis. We found that neuritogenesis was significantly suppressed on a gel substrate with an elastic modulus higher than the stiffness of *in vivo* brain. Fluorescent images of the F-actin cytoskeletal structures showed that the F-actin organization depended on the substrate stiffness. Circumferential actin meshworks and arcs were formed at the edge of the cell body on the stiff gel substrates unlike with soft substrates. The suppression of F-actin cytoskeleton formation improved neuritogenesis. The results indicate that the organization of neuronal F-actin cytoskeletons is strongly regulated by the mechanical properties of the surrounding environment, and the mechanically-induced F-actin cytoskeletons regulate neuritogenesis.

## Introduction

Cells recognize and respond to external mechanical signals in their surrounding microenvironment such as an extracellular matrix (ECM) [1]. There have been various reports into the effects of mechanical forces *in vitro*, including applied substrate strain and substrate stiffness, on cell behaviors. Various cell types such as endothelial cells, fibroblasts, and smooth muscle cells exhibit morphological changes and migration that are dependent on substrate stiffness although the preferential stiffness range is dependent on cell type [1–4]. Recent studies have revealed that neural cells including several types of neurons and glial cells, which are the main constituents of the central nervous system (CNS), respond to mechanical cues [5–8]. There have been various reports on how the mechanical properties of ECM affect neuronal development, growth, disorders and regeneration [9–12].

One example is traumatic injury to the CNS, which changes the mechanical properties of the surrounding environment. Injury to the CNS induces the formation of glial scars, which are mainly composed of glial cells that are responsible for the local immune response and wound healing processes. It has been reported that a glial scar prevents neurons from regenerating and elongating their neurites due to many obstacles including an injury environment filled with deleterious factors [10]. Although the role of the glial scar is under discussion [13], a change in its mechanical properties seems to act as a mechanical barrier to axon regeneration. As another example, devices implanted into the body become encapsulated due to a foreign body reaction. In the CNS, this can lead to loss of functionality in electrodes since a mechanical mismatch between nervous tissue and the devices induces the formation of glial scars [9]. From the above, mechanical matching between nervous tissue and a lesion or implant should be considered as regards developing neuronal regeneration and bio-interfaces.

Although there have been reports on neurite elongation and the dependence of its molecular pathways on the mechanical properties of the surrounding environment [14–21], less is known about how the mechanical properties affect the first morphological step of neurite formation. Neurite extension from a cell body is the first step in forming a functional nervous system and realizing neuronal regeneration [22–24]. When neurons generate and grow, they initially attach themselves to the surrounding ECM and sprout neurites from a spherical cell body. Neurite extension, which is differentiated in the axon and dendrites, forms the basis of proper neuronal connectivity and brain function. Morphological changes in hippocampal neurons from neurite initiation to neurite extension have been well studied *in vitro* [25, 26]. The neuronal development stages can be classified as follows. First, immature neurons with a localized bud from a cell membrane are classified as stage 1. The transformation of a bud into neurites of approximately equal lengths is classified as stage 2. Neurons that possess one neurite considerably longer than the rest are classified as stage 3. Neuritogenesis occurs during stage a 1 to 2 transition. First, the rearrangement of F-actin assembly in the bud induces the leading edge of the cell body to protrude. Next, microtubules and other components must be engaged with the F-actin assembly; specifically, F-actin bundles can facilitate microtubule elongation [23, 26]. Finally, the proper consolidation of the proteins leads to neuritogenesis. Actin and microtubules are the main components of neurites, and the proper organization of F-actin assembly occurs prior to microtubule organization in the process of neurite formation. Although a variety of neuronal shapes appear during development, the initial sprouting of neurites seems to follow the morphological criteria described above. When neurons are cultivated on a substrate *in vitro*, they initially attach themselves to the substrate and form lamellipodia and filopodia, which are a sheet-like extension of a crosslinked F-actin meshwork at the leading edge of cells and a thin protrusion of F-actin-bundles at the periphery of cells and growth cones, respectively. F-actin organization plays an important role in initiating neurites from the cell body.

Here, we investigated how the mechanical properties of the microenvironment affected neuronal morphologies and the F-actin structures of neurons during neurite initiation by preparing polyacrylamide gel substrates with the various stiffness ranging from similar to greater than brain stiffness. We hypothesized that the substrate stiffness can affect neurite initiation if injury-induced mechanical changes in the surrounding environment are related to the suppression of axonal regrowth or regeneration in the CNS. Since the F-actin cytoskeleton is an important structure for neurite initiation as described above, we focused on the organization of the F-actin cytoskeleton of neurons on gel substrates. A detailed investigation of the structures of the F-actin cytoskeleton reveals that the F-actin organization depends on the substrate stiffness, and the stiffness-dependent F-actin structures regulate neuritogenesis.

## Materials and methods

### Materials

The sources of the materials, chemicals and antibodies used in this work were as follows. Coverslips (No. 1, 18 mm x 18 mm) were purchased from Matsunami Glass, Japan. Acrylamide (AAm), N,N’-methylenebisacrylamide (Bis), and 3-(trimethoxysilylpropyl) methacrylate were purchased from Tokyo Chemical Industries, Japan. A photoinitiator, lithium phenyl-2,4,6-trimethylbenzoylphosphinate (LAP) was synthesized in accordance with a previous report [27]. Poly-*D*-lysine hydrobromide (PDL) and triton X-100 were obtained from Sigma-Aldrich. Blebbistatin (BS) and cytochalasin D (CD) were purchased from WAKO Pure Chemicals, Japan. ProLong Diamond as a mounting reagent, Sulfosuccinimidyl 6-(4'-azido-2'-nitrophenylamino)hexanoate (sulfo-SANPAH), *L*-glutamine, trypsin, glutamate, gentamycin, neurobasal medium, and B27 supplement were obtained from Thermo Fisher Scientific, USA. Mouse anti-β-III tubulin and phalloidin labeled with green fluorescence dye were purchased from R&D Systems, USA and Cytoskeleton, USA, respectively. Wistar rats were obtained from Charles River Laboratories, Japan.

### Preparation of hydrogel substrate

Hydrogel substrates were prepared using the following procedures. All cover glasses were used after overnight treatment with 0.1 M NaOH followed by O_2_-plasma treatment. The cleaned cover glasses were immersed in silane coupling solution containing 0.5% 3-(trimethoxysilylpropyl) methacrylate for 2 hours at room temperature. The silanized cover glasses were annealed at 110 ^o^C for 30 min. We prepared two types of hydrogel precursor solutions that contained 5% w/v AAm with 0.05, 0.1, 0.15, 0.2, 0.25, and 0.5% w/v Bis for soft gels and 0.5% w/v Bis for stiff gels. For photo-initiated polymerization, LAP was added as a photoinitiator to the precursor solutions at a final concentration of 1 mM. The precursor solution was dropped on the silanized coverslip and sealed with a cleaned coverslip with 11-μm-thick spacers. The substrate was irradiated with 360 nm wavelength UV light at 10 mW/cm^2^ for 10 min. After the light irradiation, the substrates were immersed in PBS and gently agitated at 50 rpm and 25 ^o^C over 3 days. For comparison with previous reports about the neuritogenesis of neurons on glass substrates, we used poly-D-lysine as adhesive molecules. To display the PDL on the surface of the hydrogel, sulfo-SANPAH was used as a crosslinker. After a 10-min UV irradiation of the sulfo-SANPAH solution on the hydrogel, 1 mg/mL of PDL was applied to the hydrogel surface.

### Cell culture

All animal experiments were approved by Biological Safety and Ethics Committee of NTT Basic Research Laboratories (approval ID 2014–04). Wistar rats (embryo day 18) were used to obtain hippocampal cells. They were used immediately after receipt, and anesthetized with a gas mixture of 1–3% isoflurane and air during preparation. Every effort was made to minimize suffering. The hippocampus was extracted from rat brain, and then treated with 2.5 mg/ml trypsin for 10 min at 37 ^o^C. The cells were then centrifuged at 1000 rpm for 5 min and triturated with a pipette. The culture was carried out with a neurobasal medium that consisted of 74 μg/ml *L*-glutamine, 25 μM glutamate, 50 μg/ml gentamycin and 2% B27 supplement. The cell suspension was applied to gel substrates with an initial cell number of 15000 cells/substrate, and cultured at 37 ^o^C and in 5% CO_2_, with saturated humidity.

### Measurement of elastic modulus of hydrogel substrates

A customized atomic force microscope (AFM) equipped on an upright optical microscope (Eclipse FN1, Nikon, Japan) was used to measure the Young’s modulus, *E*, of the gel substrates. A rectangular cantilever (BioLever mini, BL-AC40TS-C2, Olympus, Japan) with a nominal spring constant of less than 0.1 N/m was used. The loading force was determined using Hook’s law by multiplying the cantilever deflection by the spring constant calibrated using a thermal fluctuation method. The force curve measurements were examined in a 300 μm × 300 μm scan region at a maximum loading force of around 1.5 nN. *E* was estimated from the observed force-distance curves with the Sneddon’s modulation of a Hertzian contact model as a conical indenter, which is expressed as:

$$F=\frac{2\tan\alpha }{\pi }\frac{E}{\left(1-\nu^{2}\right)}\delta^{2},$$ (1)

where *F* is the loading force, *δ* is the indentation depth, α is the half angle of the conical probe of 17.5°, and *ν* is the Poisson’s ratio of the gel substrate, assumed here to be 0.5.

### Fluorescence staining

To count neurons without neurites on a polyacrylamide gel substrate, we used calcein-AM and propidium iodide (PI) to stain living and dead neurons, respectively. These fluorescent dyes were directly added to samples in a culture medium. After 30 minutes’ incubation at 37 ^o^C in a 5% CO_2_ atmosphere, the cells were observed with a fluorescent microscope. The cells were immunofluorescently stained as follows. All the samples were fixed with 4% paraformaldehyde in PBS, permeabilized with 0.5% Triton X-100 in PBS, and then blocked with a mixture of 5% NBS and 1% BSA in PBS at room temperature. After the blocking treatment, primary antibodies were applied to the cells followed by washing and incubation with appropriate secondary antibodies bound to fluorescent dyes. For F-actin staining, the cells were treated with 0.5% TritonX-100 in PBS, and then stained with phalloidin bound to fluorescent dyes. Fluorescently labeled samples were mounted with a mounting reagent. To distinguish neurons from the other hippocampal cells, neuron-specific marker β-III tubulin with anti-β-III tubulin antibody and 500 times dilution. It was a marker for neuritogenesis because it is the main component of a microtubule in neurites. The antibodies were labeled with species-specific secondary antibodies conjugated with Alexa 568 with 400 times dilution.

### Fluorescent imaging

Fluorescent images were obtained using a fluorescent microscope (Eclipse TE3000, Nikon, Japan) with a CMOS camera (ORCA flash 4.0, Hamamatsu Photonics, Japan), and a laser scanning confocal microscope (LSM510, Carl Zeiss, Germany or IX81, Olympus, Japan). The microscope setup for the spinning disc superresolution microscope (SDSRM) is based on a disk-scanning confocal microscope system, which includes an IX81 and a disc-scanning unit (IX2-DSU, Olympus, Japan). To investigate neuritogenesis and F-actin bundle formation, acquired images were analyzed with ImageJ (NIH, http://rsb.info.nih.gov/ImageJ).

### Statistical analysis

All experiments were performed using at least three independent donors and three replicate gel substrates. To obtain a statistical analysis of neuritogenesis, we classified neurons that had protrusions with a diameter less than that of the cell body as stage 1. Protrusions whose diameter exceeded that of the cell body were classified as neurites. Neurons with neurites of approximately equal length were defined as stage 2. Neurons that possessed one neurite at least twice as long as the rest were classified as stage 3. Statistical comparisons were performed using an independent t-test when filopodia density and length were compared, and one-way analysis of variance (ANOVA) with Bonferroni’s *post hoc* testing was used to make pairwise comparisons between multiple groups. The statistical significance was set at *p* < 0.05.

## Results

### Characterization of gel substrates

A hydrogel substrate with a homogeneous elastic modulus and defined thickness is required if we are to investigate how substrate stiffness affects neuritogenesis [28–32]. To characterize the gel substrate, the Young’s modulus, *E*, was measured with AFM with different crosslinker concentrations. The results were shown in Table 1. E for 0.05% BIS concentration was 1.7 × 10^2^ (± 0.3 × 10^2^) Pa, which was comparable to *in vivo* brain stiffness [12]. E for 0.1% BIS concentration was 2.2 × 10^3^ (± 0.3 × 10^2^) Pa, and E for more than 0.15% BIS concentration was 3.2 × 10^3^ (± 0.5 × 10^3^) Pa, which was one order of magnitude higher than the brain’s stiffness.

**Table 1:** Young’s modulus of polyacrylamide gels used in this study.

AAm (%)	BIS (%)	Young’s modulus (Pa)
5	0.05	1.7 × 10^2^ ± 0.3 × 10^2^
5	0.1	2.2 × 10^3^ ± 0.3 × 10^3^
5	0.15	3.8× 10^3^ ± 0.7 × 10^3^
5	0.2	3.4 × 10^3^ ± 0.6 × 10^3^
5	0.25	3.4 × 10^3^ ± 0.6 × 10^3^
5	0.5	3.2 × 10^3^ ± 0.5 × 10^3^

### Neuritogenesis on polyacrylamide gel substrates

**Fig 1:**
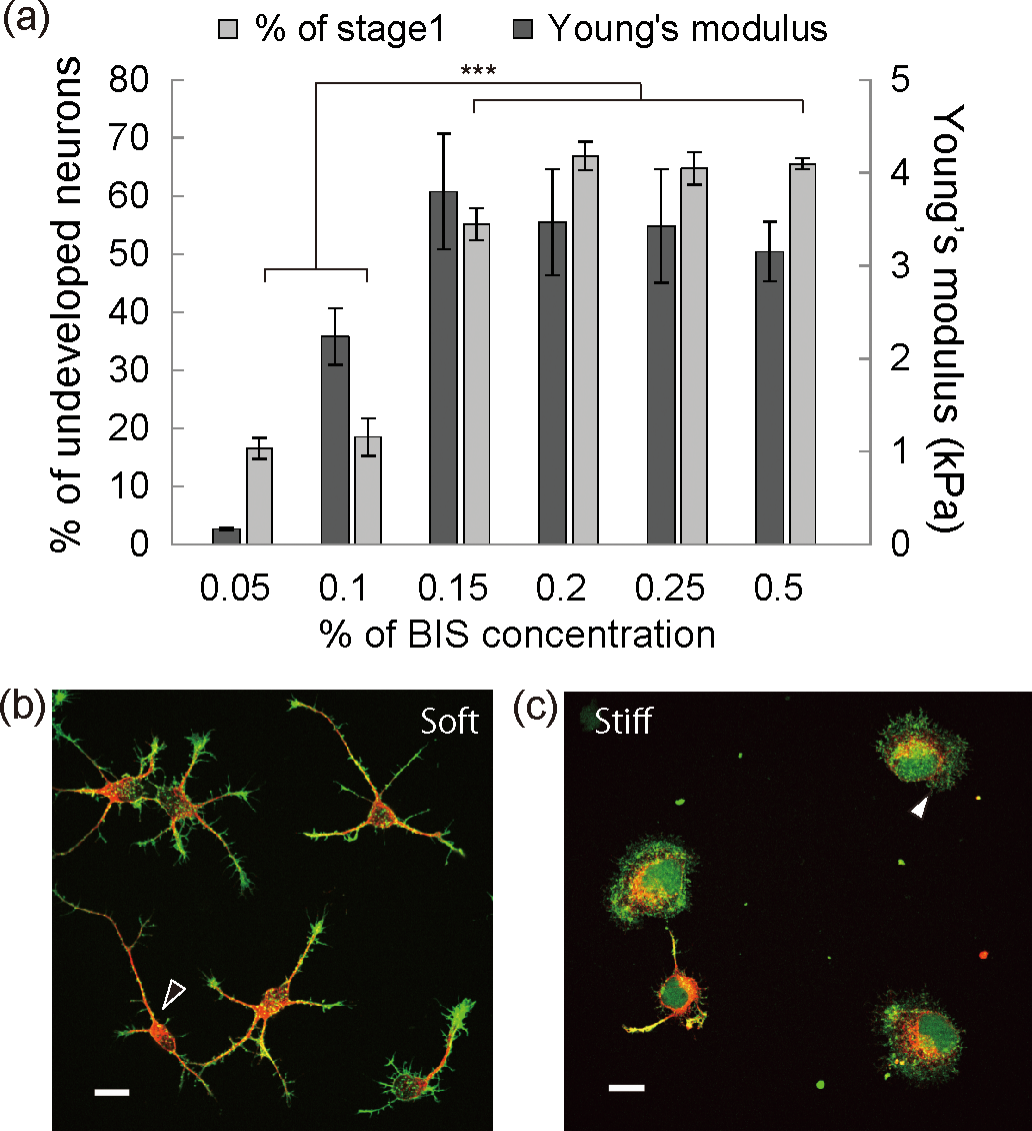
Substrate stiffness dependent neuritogenesis. (a) Dark gray bars and light gray bars indicate the Young’s moduli of the gel substrates and neurons in stage 1 as a percentage of all the neurons on the gel substrates at 26 hours after plating, respectively. (b, c) Immunofluorescent images of representative neuronal morphologies on a soft gel substrate (b) and a stiff gel substrate (c) at 20 hours after plating. Red: β-III tubulin, green: F-actin.

As described in the Introduction, neuronal developmental stages *in vitro* can be divided from neuronal morphologies [26]. To obtain a statistical analysis of neuritogenesis on the gel substrates, we defined neurons with the protrusions that were less than the diameter of the cell body as stage 1 and elongation of the protrusions equal to the diameter of the cell body as neurite initiation or neuritogenesis. We counted the number of neurons in stage 1 from fluorescent images of neurons stained with calcein-AM and PI, which allowed us to distinguish living neurons from dead ones in stage 1. Fluorescence observations showed that the cellular viability on each gel substrate was not statistically significant (S1 Fig). The neurons in stage 1 as a percentage of the total number of cells on the gel substrates are shown in Fig 1A. The graph shows that the percentage of neurons in stage 1 on stiff substrates at 26 hours after plating was 17%±1.8% for 0.05% BIS, 19%±3.2% for 0.1% BIS, 55%±2.8% for 0.15% BIS, 66%±2.5% for 0.2% BIS, 65%±2.8% for 0.25% BIS, and 66%±1.0% for 0.5%BIS. The results indicate that the neuritogenesis on the gel substrates with elastic moduli more than 3.0 kPa is significantly suppressed. To investigate the stiffness dependent suppression of neuritogenesis, we hereafter used gel substrates prepared from 0.05% BIS as the standard substrate (soft substrate) and 0.5% BIS as the stiffer gel substrate (stiff substrate). We statistically analyzed the further development of neurons on soft and stiff substrates at 20 hours after plating (S2 Fig). For the analysis, protrusions that exceeded the diameter of the cell body were classified as neurites. Neurons with neurites of approximately equal length were defined as stage 2. Neurons that possessed one neurite at least twice as long as the rest were classified as stage 3. The percentages of neurons in stage 2 were 48% ± 10% for the soft substrate and 23% ± 4% for the stiff substrate, whereas the percentages in stage 3 were 26% ± 14% for the soft substrate and 2.6% ± 3% for the stiff substrate. The differences are statistically significant. The results suggested that substrate stiffness affected neurite elongation and development as well as neuritogenesis.

To investigate neuronal morphologies, the neurons were immunofluorescently labeled with phalloidin and anti-βIII-tubulin for F-actin and microtubule, respectively. Fluorescent images of neurons on soft and stiff substrates at 20 hours after plating are shown in Fig 1B and 1C, and S3 Fig. respectively. On soft substrates, fluorescent images revealed the co-existence of neurons with and without red fluorescence from anti-βIII-tubulin, indicating the formation of neurites (Fig 1B). The neurons had thin and short protrusions with green fluorescence from phalloidin, indicating F-actin fibers (Fig 1B, black arrow head). Compared with the soft substrates, fewer neurons had protrusions with red fluorescence, and F-actin meshworks with green fluorescence were formed at the cell periphery on stiff substrates (Fig 1C, white arrow head). These fluorescent images indicated that the stiffening of the surrounding area induces neuronal morphological changes, especially F-actin organization.

### F-actin cytoskeleton structure

**Fig 2:**
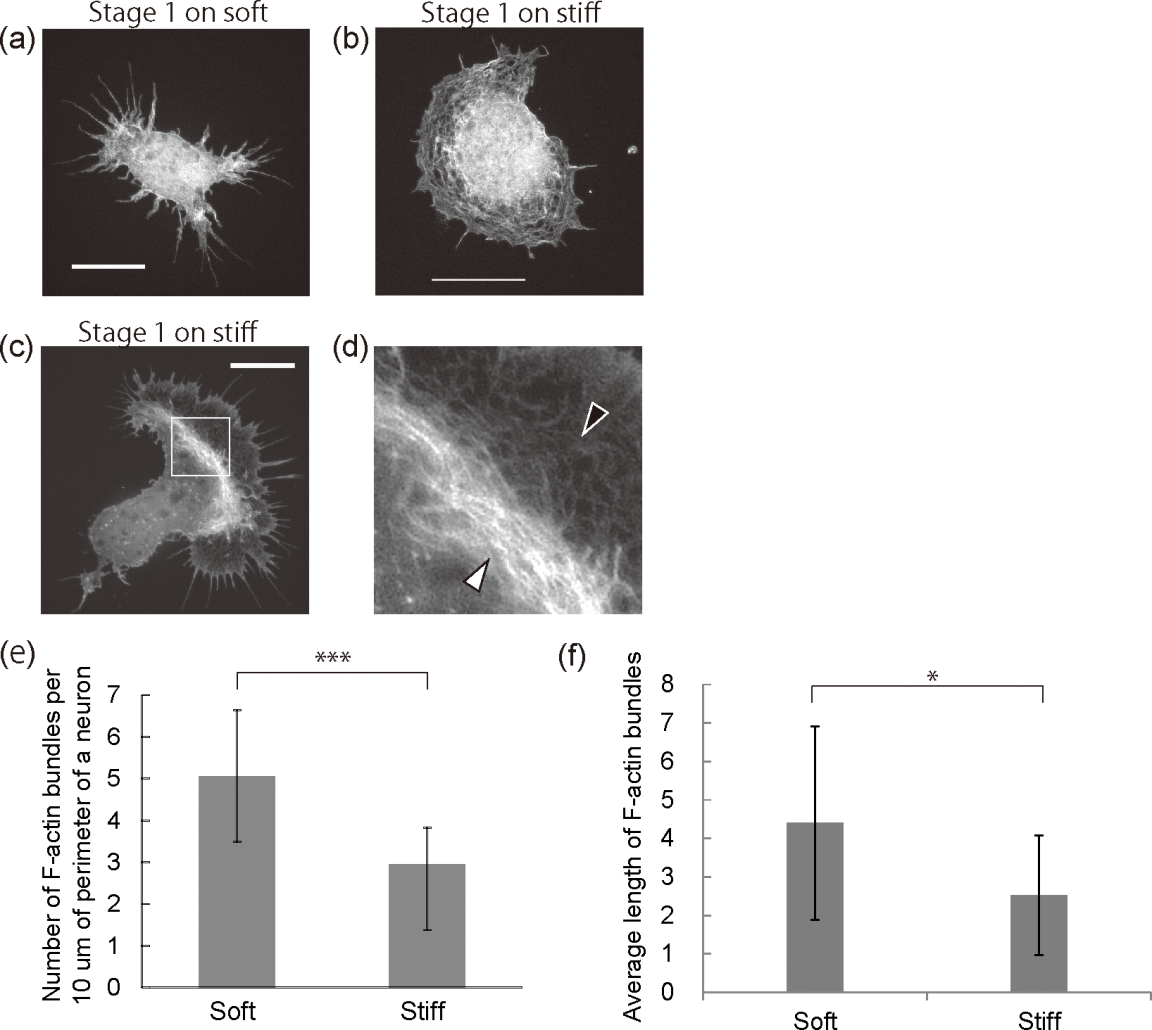
Organization of F-actin cytoskeleton on gel substrates. (a, b) Fluorescent images of representative neurons in stage 1 on the soft substrates (a) and stiff substrates (b). (c) A fluorescent image of a representative neuron with F-actin arcs. (d) A magnified image of the boxed area in (c). All neurons in (a-d) were fixed at 20 hours after plating and stained with phalloidin. All scale bars are 10 mm. (e) Number of F-actin bundles per 10 mm of neuron perimeter after 20 hours of cultivation. (n = 40, number of cells per group, ***p<0.001) (f) Average length of F-actin bundles after 20 hours of cultivation (n = 444, number of filopodia from replicating three gel substrates per group, *p<0.05).

To assess whether F-actin organization induced by substrate stiffness is involved in neurite initiation, the F-actin structures were observed in detail with a spinning disk superresolution microscope (SDSRM) [33]. Fig 2A–2D show representative fluorescent images of neurons on polyacrylamide gel substrates. At developmental stage 1, neurons on stiff substrates had larger areas of circumferential F-actin meshworks as shown in Fig 2C compared with the soft substrates shown in Fig 2A. Some neurons on the stiff substrates had a F-actin structure with two distinct parts (Fig 2C and 2D). One part indicated by a black arrowhead consisted of F-actin meshworks and the other indicated by a white arrowhead consisted of condensed F-actin meshworks, known as F-actin arcs. F-actin arcs were not observed on the soft substrates with our experimental setup. At developmental stage 2, neurons on the stiff substrates had larger growth cones at the tips of neurites and larger areas of F-actin meshworks than soft substrates (S4 Fig). On the other hand, fewer F-actin bundles sprouted from the leading edge of a cell body and neurites on the stiff substrates. The fluorescent images showed that F-actin bundle formation was suppressed on stiff substrates. To analyze the density and length distribution of F-actin bundles statistically, we counted the number and measured the length of F-actin bundles protruding from the perimeter of neurons on a soft or a stiff substrate. The graph in Fig 2E, shows the number of F-actin bundles per 10 μm at the perimeter of a neuron. Neurons on stiff substrates have F-actin bundles with significantly lower densities than those on soft substrates. In addition, the average length of the F-actin bundles on stiff substrates (2.5±1.6 μm) are significantly shorter than those on soft substrates (4.4±2.5 μm) (Fig 2F). It has been reported that the prevention of filopodia formation suppressed neuritogenesis because filopodia can guide microtubule elongation during neuritogenesis [22, 23]. Our results imply that the suppression of F-actin bundle formation on stiff substrates is involved in neuritogenesis. Moreover, stiffness of the gel substrates induced a change in the cytoskeletal F-actin organization including the formation of a larger area of F-actin meshworks and F-actin arcs. Therefore, we hypothesized that F-actin organization induced by substrate stiffness can play a role in regulating neuritogenesis.

### Blebbistatin treatment

**Fig 3:**
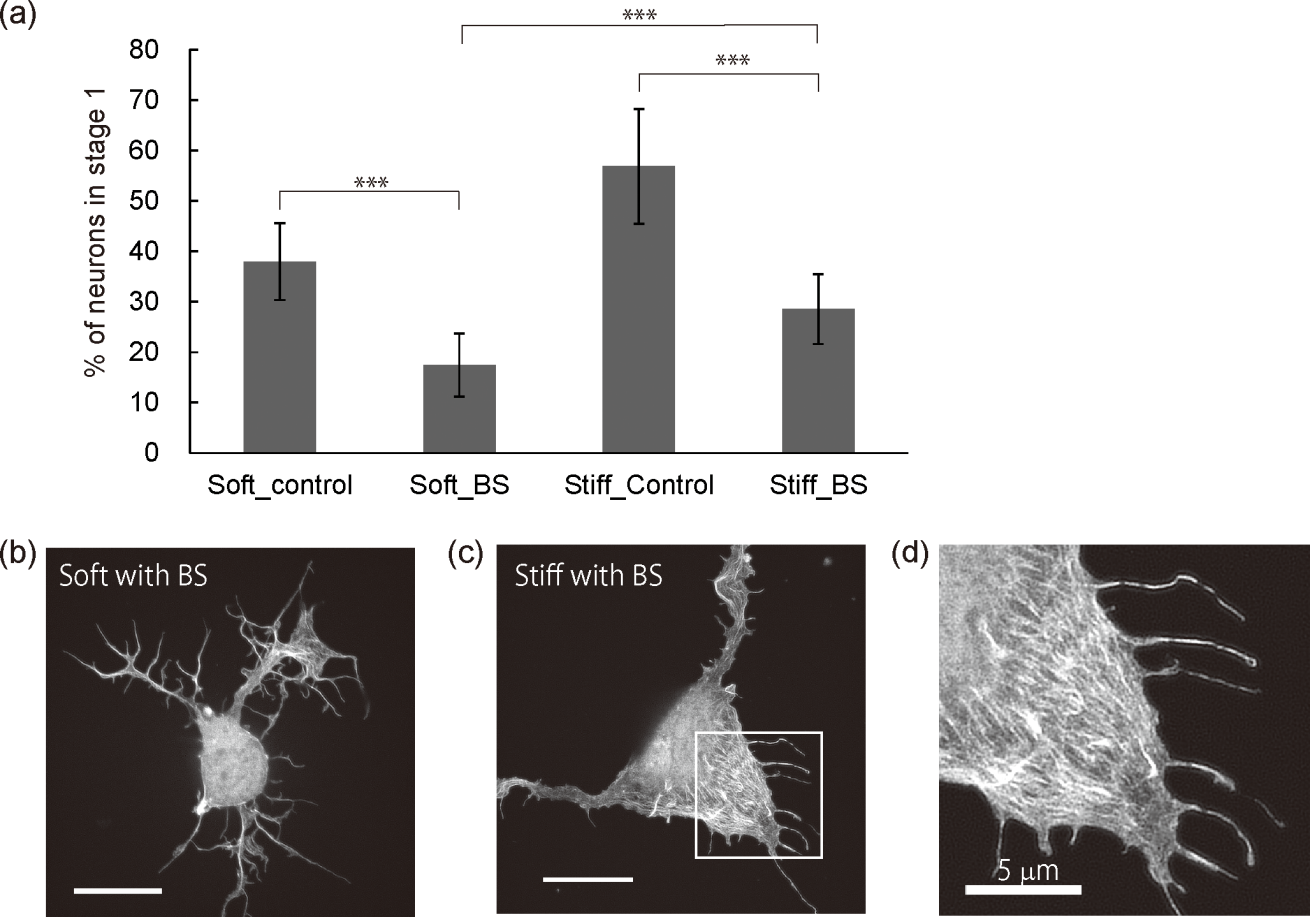
Effects of blebbistatin on neuritogenesis of neurons on gel substrates. All neurons were exposed to 100 mM BS or DMSO for 20 hours. (a) Neurons in stage 1 as a percentage of all the neurons on the gel substrates with/without BS treatment. (n > 300 cells per group, *p<0.001 by one-way ANOVA with Bonferroni post hoc test) (b, c) Fluorescent images of representative neurons on soft substrates (b) and on stiff substrates (c). (d) A magnified image of the boxed area in (c). All scale bars except for (d) are 10 mm.

We investigated the way in which the organization of the F-actin cytoskeleton, and in particular the circumferential F-actin arcs and the suppression of the F-actin bundles, are associated with neuritogenesis. We used blebbistatin (BS), which is known to be a myosin II inhibitor and inducer of filopodia formation [34, 35]. Since myosin II activity contributes to the compaction of F-actin meshworks and the consequent formation of F-actin arcs, BS treatment can suppress F-actin arc formation. BS was exposed at a concentration of 100 μM, which was sufficient to attenuate the interaction between F-actin and myosin II, immediately after cell plating [34]. The neuritogenesis of neurons on gel substrates after BS exposure was analyzed statistically by counting the number of neurons in stage 1 at 20 hours after plating (Fig 3A). As control experiments, neurons on gel substrates were exposed to dimethyl sulfoxide (DMSO), which did not affect neuritogenesis or the F-actin structures (S5 Fig). The graph shows that the percentage of neurons in stage 1 on both the soft and stiff substrates decreased significantly after the BS (from 36% to 17% for soft substrates, and from 55% to 29% for stiff substrates). Although the BS treatment improved neuritogenesis, the percentage of neurons in stage 1 on stiff substrates was higher than that on soft substrates, indicating the suppression of neuritogenesis. To investigate BS-induced neurite protrusions, an SDSRM was used for a detailed observation of the F-actin structures after BS treatment on the stiff substrate (Fig 3B and 3C). We could not find significant structural changes of F-actin organization on the soft substrates after BS treatment when compared with neurons without BS exposure (Fig 2A). On the other hand, on stiff substrates, although the circumferential actin arcs disappeared, F-actin meshworks were observed at the edge of a cell body (Fig 3C). The F-actin meshworks were segmented, and the widely spread growth cones observed before BS treatment (Fig 2D) were barely seen. Parallel arranged F-actin organization was observed in the segmented F-actin area (Fig 3D). These F-actin structures were not observed on the soft substrate. These results indicate that F-actin structures induced by the attenuation of myosin II activity are associated with neuritogenesis, although the myosin II activity is strongly involved in neurite initiation.

### Cytochalasin D treatment

**Fig 4:**
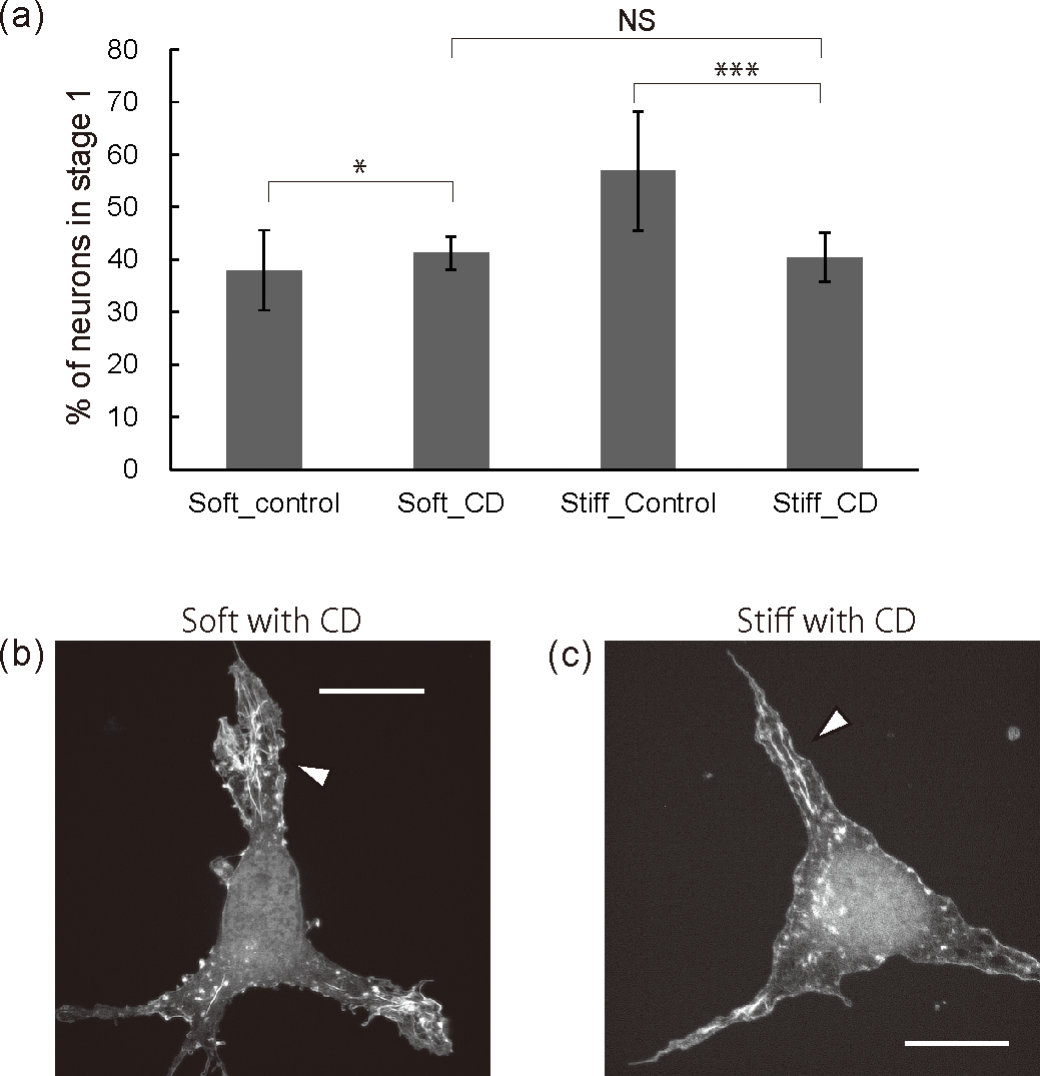
Effects of cytochalasin D on neuritogenesis of neurons on gel substrates. All neurons were exposed to 100 nM CD or DMSO for 20 hours. (a) Neurons in stage 1 as a percentage of all the neurons on the gel substrates with/without CD treatment. (n>300 cells per group, *p<0.05, ***p<0.001 by one-way ANOVA with Bonferroni *post hoc* test) (b, c) Fluorescent images of representative neurons on soft substrates (b) and on stiff substrates (c). All scale bars are 10 μm.

Although the exposure of neurons on gel substrates to BS revealed that F-actin arcs at the leading edge of a cell body suppressed neuritogenesis, it remained unknown whether the other aspects of F-actin organization, which was observed after the BS treatment, were related to neuritogenesis. Therefore, we attempted to completely suppress F-actin organization by treating neurons with cytochalasin D (CD), which induces the disruption of actin filaments and prevents actin polymerization by binding to the barbed end. CD was exposed at a concentration of 100 nM, which was sufficient to disrupt the F-actin structures at the leading edge of the cell body, immediately after cell plating [22]. To assess whether CD treatment affected neuritogenesis, we counted the neurons without neurites on the hydrogel substrates (Fig 4A). The control results were the same as those in Fig 3A. The exposure of CD to neurons on soft substrates significantly impaired neuritogenesis whereas it significantly improved neuritogenesis on stiff substrates (36% to 41% for soft substrates and 55% to 40% for stiff substrates). In addition, the percentages of the soft and stiff substrates after CD treatment had no statistical significance. To investigate the way in which CD treatment affected the F-actin organization of the neurons on the soft and stiff substrates, the structure of the F-actin cytoskeleton was observed in detail with an SDSRM (Fig 4B and 4C). Neurons on both soft and stiff substrates had similar morphologies after CD treatment. There was no F-actin bundle formation and no meshwork on the gel substrates, indicating that the CD concentration in Fig 4 was sufficient to disrupt the F-actin structures at the leading edge of the cell body. The suppression of F-actin organization induced the suppression of neuritogenesis on the soft substrates but the acceleration of neurite initiation on the stiff substrates. As F-actin bundles can accelerate neuritogenesis, the suppression of F-actin bundle formation on the soft substrates resulted in the suppression of neuritogenesis. On the other hand, whether or not CD treatment improved neuritogenesis on the stiff substrates remains controversial. CD-induced changes in the F-actin organization on the stiff substrates consisted of the disruption of F-actin meshworks and arcs at the leading edge as well as the suppression of F-actin bundles. This suggests that F-actin structures formed on the stiff substrates help prevent the suppression neuritogenesis.

## Discussion

It has been reported that the mechanical properties of ECM in various tissues varied during several events such as external stimuli, aging, and diseases. With respect to the CNS, understanding the relationship between ECM mechanical properties and neural functions is an important issue in the field of tissue engineering and neuroscience. This study demonstrates the effects of substrate stiffness on the neuritogenesis of hippocampal neurons because neuritogenesis is the first step towards development and regeneration. Also, we investigated structure of the F-actin cytoskeleton as proper arrangements of the F-actin assembly are required prior to organization of the other proteins related to neurites.

To accomplish this, we employed a polyacrylamide gel as a substrate with a mechanical property ranging from similar to a hippocampus to a stiffer one. As an adhesion molecule, we used PDL which can act as a non-selective focal adhesion activator [19]. Previous study reported that adhesion molecules derived from ECM proteins such as laminin, fibronectin and collagen, initiate integrin-mediated cell binding via the formation of focal adhesions, which is a key structure as regards regulating mechanotransduction and activating signaling pathway of neuritogenesis [36]. For example, laminin can facilitate neurite initiation and axonal outgrowth compared with fibronectin [18, 19]. Moreover, laminin can rescue the neuritogenesis of neurons whose neurite initiation is genetically inhibited whereas collagen and fibronectin have no effect [23, 24]. These findings indicate that distinct ECM proteins activate distinct signaling pathways related to neuritogenesis. On the other hand, PDL modulates cell adhesion via an electrostatic interaction between a negatively charged cell membrane and a positively charged PDL. The PDL-mediated non-selective activation of focal adhesion allows us to eliminate the possibility that specific activation of a certain signaling pathway induced by ECM proteins affects suppression or acceleration of neritogenesis.

We found that a stiff substrate with an elastic modulus exceeding 3.0 kPa changed the neuronal morphologies and organization of the F-actin cytoskeleton, and suppressed neuritogenesis. The further development of neurons from stage 2 to 3 was suppressed on the stiff substrate. Noteworthy features of the morphologies of neurons on the stiff substrates were that they had fewer F-actin bundles and the formation of more circumferential F-actin meshworks and arcs at the leading edge. According to a previous report stating that sprouts of F-actin bundles are required for neuritogenesis, the suppression of F-actin bundle formation on stiff substrates prevents microtubules from invading and protruding from the leading edge [22, 37–39]. Together, F-actin meshworks and arcs on stiff substrates appeared to suppress neurite protrusions. Regarding the further development from stage 2 to 3, the neurite elongation at the leading edge of the growth cones can be suppressed by the stiffer substrate-induced F-actin structures.

To assess our assumption that stiffer substrate-induced F-actin organization plays a critical role in neuritogenesis, we treated neurons on polyacrylamide gel substrates with BS to inhibit the formation of actin arcs. The disruption of F-actin arcs is observed on stiff substrates after the BS treatment. It is known that neurons, which genetically lack the ability to form filopodia, fail to initiate neurites, and they form F-actin arcs at the leading edge [22, 23]. In the report, BS treatment disrupts the F-actin arcs and rescues neuritogenesis. These results are similar to ours. Although we must analyze protein expression if we are to discuss the relationship between previous reports and our results, substrate stiffness may regulate protein expression relating to cytoskeletal organization. Also, BS treatment accelerates F-actin bundle formation and improves neuritogenesis regardless of the substrate stiffness. Since an F-actin bundle assists microtubule protrusion at the leading edge of the cell body as described above, the acceleration of F-actin bundle formation can help to improve neuritogenesis.

After BS treatment, neuritogenesis on stiff substrates was suppressed compared with that on soft substrates even though the formation of actin arcs was prevented and F-actin bundle formation was accelerated. This suggests that the F-actin structure at the leading edge of neurons on the stiff substrates after BS treatment prevents microtubule protrusion. To prevent the F-actin organization at the leading edge, neurons on the gel substrates were treated with cytochalasin D. After the CD treatment, the percentages of neurons in stage 1 on the soft and stiff substrates are similar, indicating the suppression of neuritogenesis on soft substrates and the improvement on stiff substrates. Fluorescent images revealed that the structures of F-actin cytoskeletons on stiff substrates were similar to those on soft substrates after the CD treatment. The suppression of neuritogenesis on the soft substrates strongly supports the view that F-actin bundles play a beneficial role in sprouting neurites. On the other hand, neuritogenesis on the stiff substrates was improved even though the formation of the F-actin bundles was suppressed. This supports the idea that the F-actin meshworks and arcs prevent microtubules from sprouting from the leading edge. It is known that the elongation process in the growth cones takes place preferentially where the actin meshwork is unstable, whereas the stable actin meshwork tends to impair microtubule protrusion [38, 39].

**Fig 5:**
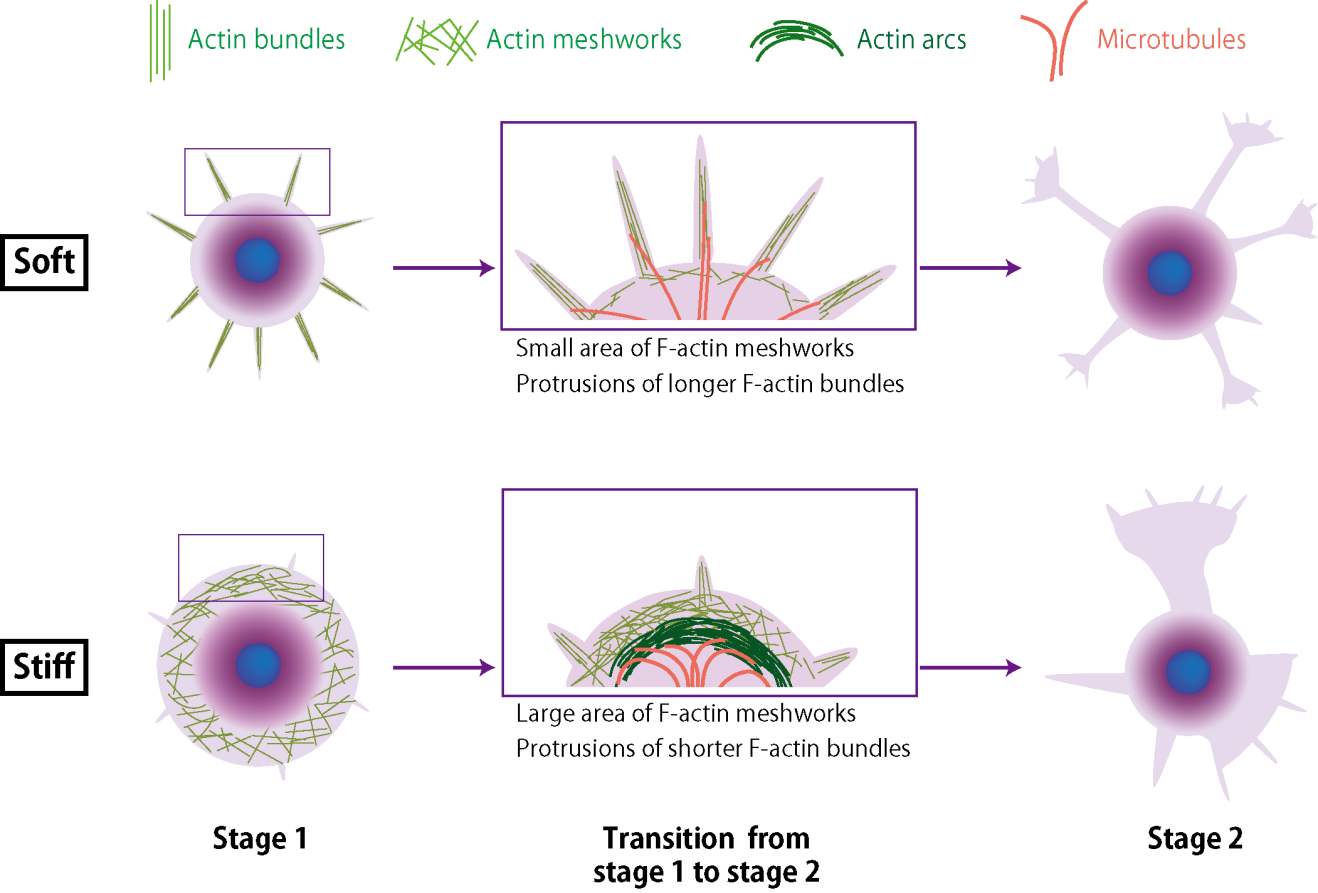
Schematic representation of proposed mechanism of neuritogenesis on gel substrates.

We propose a neuritogenesis mechanism that is dependent on substrate stiffness as shown in Fig 5. F-actin cytoskeletons such as F-actin meshworks and arcs at the leading edge of the cell body were preferentially organized on the stiff substrates, whereas F-actin bundle formation was suppressed. The F-actin structures on the stiff substrates are obstacles to the protrusion of microtubules from the leading edge of the cell body. Therefore, on a stiff substrate, the disruption of F-actin organization by CD had an effect on neuritogenesis even though F-actin bundle formation was suppressed.

## Conclusions

We investigated how substrate stiffness affected neuritogenesis. The high-resolution microscopy observations showed that stiff substrates significantly suppressed neuritogenesis due to their stronger myosin II-based contractility and stabilized the F-actin cytoskeleton. To our knowledge, this is the first observations regarding the organization of neuronal F-actin cytoskeleton regulated by the mechanical properties of the surrounding environment. The stiffness-induced F-actin structures played a key role in regulating neuritogenesis. Our findings suggest that the suppression of neuronal regeneration at a glial scar could be influenced in part by a stiffening microenvironment, which suppresses neuritogenesis and development to the later stage. Further investigations are required to understand the neuronal development because various proteins are properly associated and dissociated during neurite development. Of the several processes, the interaction of multiple proteins with F-actin assembly can be important as regards protrusions maturing into neurites and developing neurons from stage 2 to stage 3, corresponding to axon formation.

In this study, we qualitatively discuss the circumferential F-actin meshworks and arcs because we did not observe the structures on the soft substrates with our experimental setup. It is known that the low contractile force at the interface between a cell and a substrate suppresses the formation of stable adhesion and cytoskeleton assembly at the interface [1]. This is why no F-actin meshworks were observed on the soft substrates. We must investigate the dynamics of F-actin structures and other proteins such as microtubules at the leading edge during neuritogenesis by time-lapse imaging to verify our proposed model. The 3D cultivation of neurons on artificial platforms such as extracted ECM, collagen, and biocompatible polymers has been reported [40]. However, less is known about how the mechanical properties of ECM affect neuronal development in 3D cultivation. Although our experimental methods cannot be applied to 3D experiments due to the toxicity of gel monomers, our findings suggest that ECM stiffness can affect the F-actin structure of neurons in 3D cultivation. This study provides an insight not only for developing a scaffold for neuronal regeneration, but also for designing a compliant interface between tissue and a device such as a brain-machine interface.

## Supporting information

S1 Fig Cellular viability on gel substrates. The cellular viability on each gel substrate was not statistically significant.
(TIF)

S2 Fig Stacked histogram of percentage of neurons in stage. (n>200 cells per group, ***p<0.001 by one-way ANOVA with Bonferroni *post hoc* test)
(TIF)

S3 Fig Immunofluorescent images of representative neuronal morphologies on gel substrates. The substrates were prepared from gel precursor solutions containing 5%AAm and 0.1% BIS (a), 0.15% (b), 0.2% (c), and 0.25% BIS (d). Red: β-III tubulin, green: F-actin. Scale bars are 20 μm.
(TIF)

S4 Fig Fluorescent images of F-actin cytoskeleton of neuron in stage 2 on the soft substrate (a) and the stiff substrate (b).
(TIF)

S5 Fig Neurons in stage 1 as a percentage of all the neurons on the gel substrates with/without DMSO exposure. (n > 300 cells per group).
(TIF)
