# Supplementary figures and images for: Regulation of neuritogenesis in hippocampal neurons using stiffness of extracellular microenvironment
Source: PLoS One. 2018 Feb 6;13(2):e0191928. doi: 10.1371/journal.pone.0191928 (PMC5800654; doi:10.1371/journal.pone.0191928)

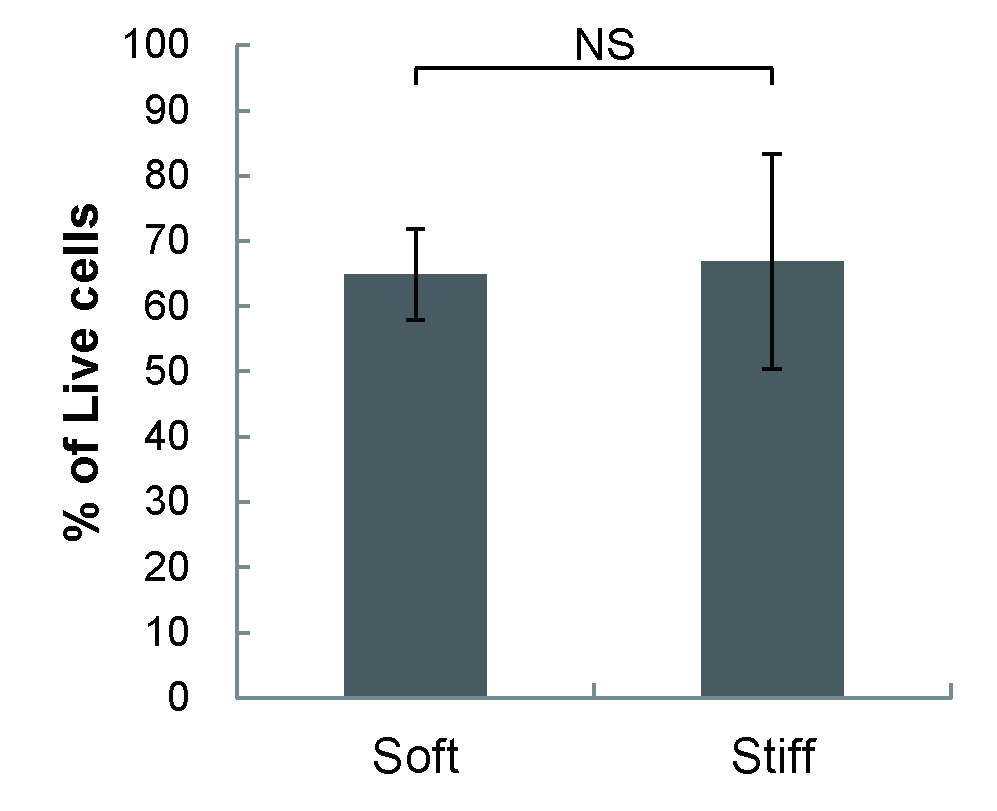

Supplement: S1 Fig — The cellular viability on each gel substrate was not statistically significant. (TIF) [file pone.0191928.s001.tif]

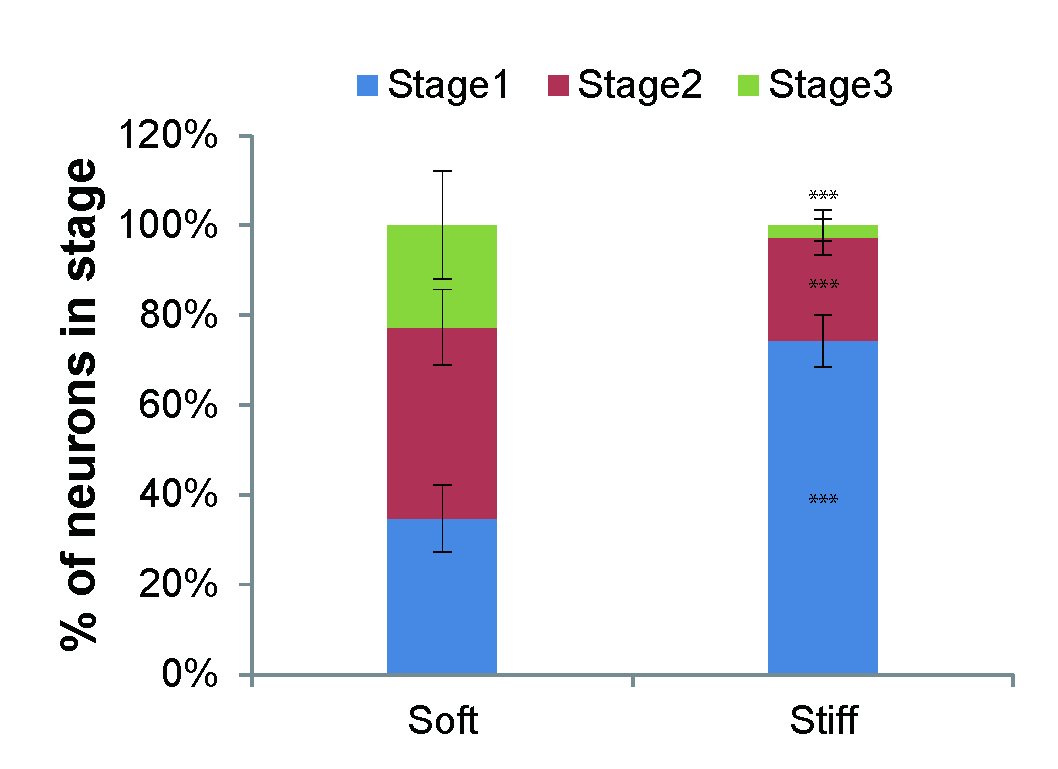

Supplement: S2 Fig — (n>200 cells per group, ***p<0.001 by one-way ANOVA with Bonferroni post hoc test) (TIF) [file pone.0191928.s002.tif]

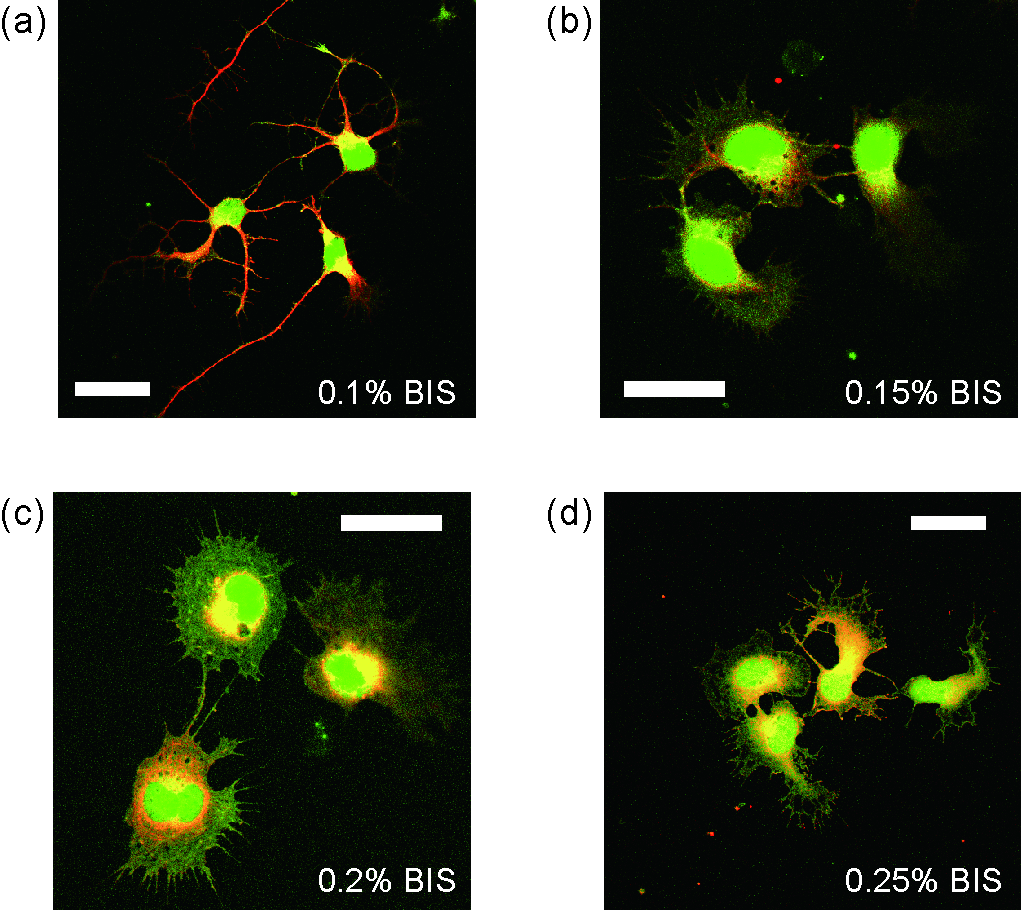

Supplement: S3 Fig — The substrates were prepared from gel precursor solutions containing 5%AAm and 0.1% BIS (a), 0.15% (b), 0.2% (c), and 0.25% BIS (d). Red: β-III tubulin, green: F-actin. Scale bars are 20 μm. (TIF) [file pone.0191928.s003.tif]

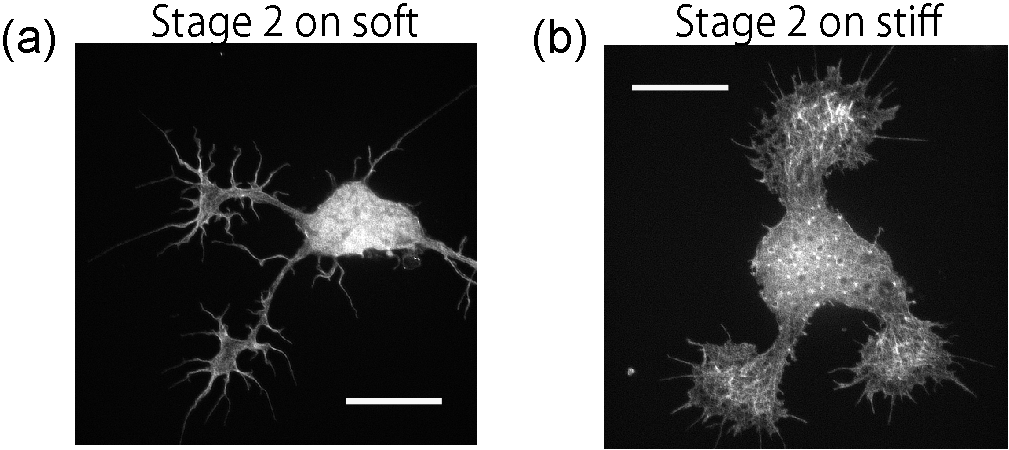

Supplement: S4 Fig — Fluorescent images of F-actin cytoskeleton of neuron in stage 2 on the soft substrate (a) and the stiff substrate (b). (TIF) [file pone.0191928.s004.tif]

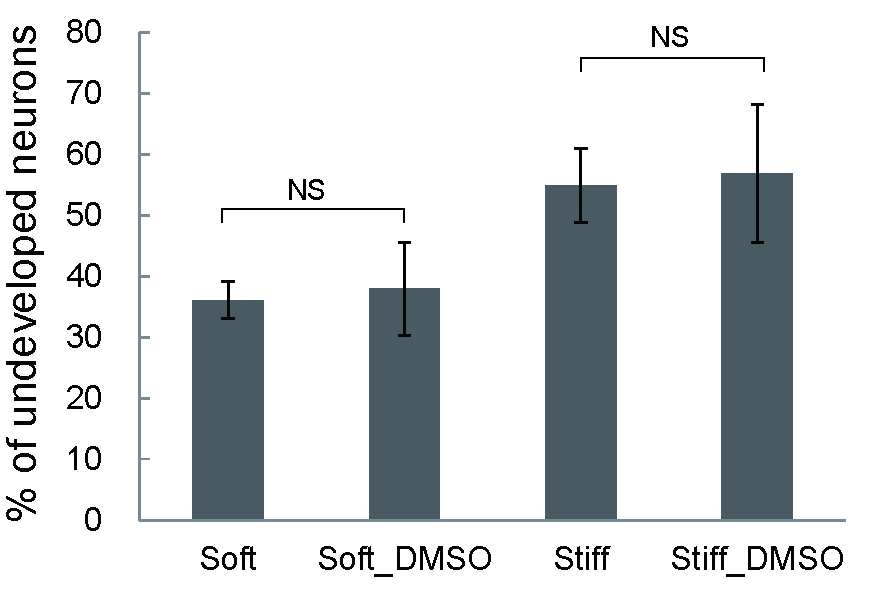

Supplement: S5 Fig — (n > 300 cells per group). (TIF) [file pone.0191928.s005.tif]
